# Non-Homologous End-Joining Pathway Genotypes Significantly Associated with Nasopharyngeal Carcinoma Susceptibility

**DOI:** 10.3390/biomedicines11061648

**Published:** 2023-06-06

**Authors:** Chia-Wen Tsai, Liang-Chun Shih, Wen-Shin Chang, Che-Lun Hsu, Jie-Long He, Te-Chun Hsia, Yun-Chi Wang, Jian Gu, Da-Tian Bau

**Affiliations:** 1 Graduate Institute of Biomedical Sciences, China Medical University, Taichung 404333, Taiwan; 2 Terry Fox Cancer Research Laboratory, Department of Medical Research, China Medical University Hospital, Taichung 404332, Taiwan; 3 Department of Otorhinolaryngology, China Medical University Hospital, Taichung 404332, Taiwan; 4 Department of Post-Baccalaureate Veterinary Medicine, Asia University, Taichung 413305, Taiwan; 5 Department of Epidemiology, The University of Texas MD Anderson Cancer Center, Houston, TX 77030, USA; 6 Department of Bioinformatics and Medical Engineering, Asia University, Taichung 413305, Taiwan

**Keywords:** DNA repair, genotype, nasopharyngeal carcinoma, non-homologous end-joining, polymorphism

## Abstract

Defects in the non-homologous end-joining (NHEJ) DNA repair pathway lead to genomic instability and carcinogenesis. However, the roles of individual NHEJ genes in nasopharyngeal carcinoma (NPC) etiology are not well-understood. The aim of this study was to assess the contribution of NHEJ genotypes, including *XRCC4* (rs6869366, rs3734091, rs28360071, rs28360317, rs1805377), *XRCC5* (rs828907, rs11685387, rs9288518), *XRCC6* (rs5751129, rs2267437, rs132770, rs132774), *XRCC7* rs7003908, and *Ligase4* rs1805388, to NPC risk, with 208 NPC patients and 416 controls. Genotype–phenotype correlations were also investigated by measuring mRNA and protein expression in adjacent normal tissues and assessing the NHEJ repair capacity in blood lymphocytes from 43 NPC patients. The results showed significant differences in the distributions of variant genotypes at *XRCC4* rs3734091, rs28360071, and *XRCC6* rs2267437 between the cases and controls. The variant genotypes of these three polymorphisms were associated with significantly increased NPC risks. NPC patients with the risk genotypes at *XRCC6* rs2267437 had significantly reduced expression levels of both mRNA and protein, as well as a lower NHEJ repair capacity, than those with the wild-type genotype. In conclusion, *XRCC4* rs3734091, rs28360071, and *XRCC6* rs2267437 in the NHEJ pathway were associated with NPC susceptibility. XRCC6 *rs2267437* can modulate mRNA and protein expression and the NHEJ repair capacity.

## 1. Introduction

Nasopharyngeal carcinoma (NPC) is a rare type of cancer with a unique geographical and ethnic distribution, occurring primarily in East Asia and Southeast Asia, especially in Southern China. East Asia has by far the highest age-standardized incidence rate of NPC, at 5.61 per 100,000 population, followed by Southeast Asia with 1.95 per 100,000 population. In contrast, central Latin America has a much lower incidence rate of only 0.23 per 100,000 population [1,2]. With the largest population and a high NPC incidence rate, China alone accounts for about 50% of new NPC cases reported worldwide each year, making it a significant healthcare concern [3]. Clinically, NPC is known for its high degree of malignancy and tendency for early lymph node metastasis [4], leading to a poor prognosis [5]. Despite advancements in medical imaging, chemotherapy, and radiotherapy technology, distant metastasis and/or local recurrence still occur in 20–30% of NPC patients, particularly those with advanced disease [4,5,6,7]. Therefore, it is crucial to identify useful biomarkers that can serve as early detection and prediction tools for identifying high-risk individuals.

Low-penetrance susceptibility genes and environmental factors are believed to play an important role in initiating and progressing tumorigenesis. Polymorphic defects in the DNA repair can undermine the network that maintains genomic stability. The most deleterious type of DNA damage is double-strand breaks (DSBs), which can result in the loss of physical integrity and information content in both strands [8]. Two important pathways for repairing DSBs induced by endogenous and exogenous carcinogens are homologous recombination (HR) and non-homologous end-joining (NHEJ). HR involves copying the missing information from an undamaged homologous chromosome during the transition from S to G2 phases of the cell cycle, while NHEJ operates during all phases of the cell cycle. NHEJ processes the broken DNA termini to make them compatible and then seals them by ligation. Notably, NHEJ is the predominant sub-pathway for DSB repair in human cells [9]. Several proteins that play crucial roles in the NHEJ pathway have been identified, including DNA Ligase4, XRCC4, XRCC6 (Ku70), XRCC5 (Ku80), and XRCC7 (DNA-dependent protein kinase, DNA-PK) [10,11]. Whenever a DSB is formed and detected, the heterodimers of Ku80 (XRCC5) and Ku70 (XRCC6) recruit the DNA-PK (XRCC7) core subunit to the DSBs, forming an active DNA-PK complex that is essential for the progression of the NHEJ repair.

An earlier pilot study reported that a single-nucleotide polymorphism (SNP), rs5751129, in the *Ku70* promoter region was associated with NPC risk [12]. However, the contributions of SNPs in other essential NHEJ genes to NPC susceptibility are still lacking. In this study, we aimed to investigate the impact of NHEJ genotypes on NPC susceptibility, as well as to examine the correlation between NHEJ genotypes and the mRNA and protein expression levels of NHEJ genes. Moreover, we assessed the NHEJ capacity in NPC patients based on their genotypes. To the best of our knowledge, this is the most comprehensive assessment of the relationship between NHEJ genotypes and NPC susceptibility.

## 2. Materials and Methods

### 2.1. Study Population

A total of 208 NPC patients were recruited from the Department of General Surgery at China Medical University Hospital in Taiwan. The patients voluntarily participated, completed a self-administered questionnaire, and provided peripheral blood samples. Non-cancer controls were matched to cases in a 2:1 ratio by gender, age (±5 years), and behavioral habits (smoking, alcohol consumption, and betel quid chewing). The exclusion criteria for controls included previous malignancy, metastasized cancer of other or unknown origin, and any genetic or familial diseases. Information on the history and frequency of smoking habits, alcohol consumption, and betel quid chewing was collected through the same self-reported questionnaire as the cases. “Ever” was defined as more than twice a week for at least one year. These behavioral habits were quantitatively evaluated and classified as categorical variables. The study was approved and supervised by the Institutional Review Board of the China Medical University Hospital (DMR101-IRB1-306).

### 2.2. Genotyping Methodologies for NHEJ Genes

**Figure 1:**
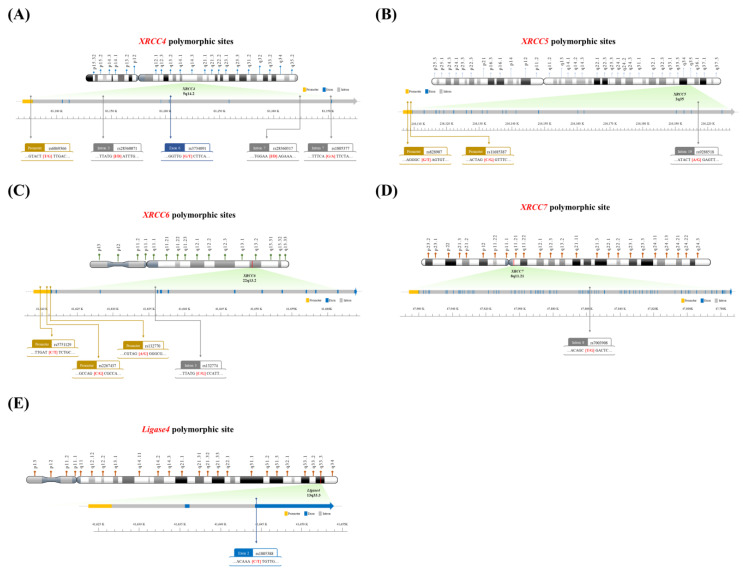
Maps of the investigated polymorphic sites in NHEJ genes: (**A**) *XRCC4,* (**B**) *XRCC5,* (**C**) *XRCC6,* (**D**) *XRCC7,* and (**E**) *Ligase4*.

The genomic DNA of each NPC patient was extracted from the peripheral blood using the QIAamp Blood Mini Kit (Blossom, Taipei, Taiwan) and stored in aliquots, as previously described [13,14]. Table 1 summarizes the information on the polymorphic sites, paired forward and reverse primers, corresponding restriction enzymes, and the resulting contigs after enzyme digestion, as well as published references [12,15,16,17,18,19]. Figure 1 shows the locations of the investigated NHEJ polymorphic sites.

### 2.3. mRNA Expressions of XRCC4 and XRCC6 Genes

To assess the correlations between NHEJ putative high-risk genotypes and gene expression, we extracted total RNA from surgically resected adjacent normal tissues of 43 NPC patients using Trizol Reagent (Invitrogen, Carlsbad, CA, USA) and measured *XRCC4* and *XRCC6* mRNA levels using real-time quantitative RT-PCR, as previously described [12]. GAPDH was used as an internal quantitative control. The forward and reverse primers for the amplification of *XRCC4* mRNA were 5′-AGCAGCCGCTATTACCGTATCTT-3′ and 5′-GTGCCAGTGTCATCATCAAATCG-3′, respectively, for *XRCC6* mRNA were 5′-CGATAATGAAGGTTCTGGAAG-3′ and 5′-CTGGAAGTGCTTGGTGAG-3′, respectively, and for GAPDH mRNA were 5′-GAAATCCCATCACCATC-TTCCAGG-3′ and 5′-GAGCCCCAGCCTTCTCCATG-3′, respectively. The results were expressed as the mean mRNA expression from triplicate measurements normalized against GAPDH as an internal control, with distilled water serving as a blank control.

### 2.4. Protein Expressions of XRCC4 and XRCC6 Genes

The adjacent normal tissues were homogenized in radio-immunoprecipitation assay (RIPA) lysis buffer obtained from Upstate Biotechnology Inc. (Lake Placid, NY, USA). The homogenates were then centrifuged at 10,000× *g* for 30 min at 4 °C, and the supernatants were used for Western blotting, as previously published [12]. Briefly, samples were denatured at 95 °C for 10 min and then separated on a 10% sodium dodecyl sulfate polyacrylamide gel electrophoresis (SDS-PAGE) gel. The separated proteins were transferred to a nitrocellulose membrane (BioRad Laboratories, Hercules, CA, USA). The membrane was blocked with 5% non-fat milk and incubated at 4 °C overnight with mouse monoclonal anti-human XRCC4 and XRCC6 antibodies (1:1000; BD Transduction Laboratories; BD Biosciences, Franklin Lakes, NJ, USA). The membrane was then incubated with the corresponding horseradish peroxidase-conjugated goat anti-mouse IgG secondary antibody (Chemicon, Temecula, CA, USA) at room temperature for 1 h. After the reaction with enhanced chemiluminescence (ECL) solution (Amersham, Arlington Heights, IL, USA), the bound antibody was visualized using a chemiluminescence imaging system (Syngene, Cambridge, UK). Finally, the blots were incubated at 56 °C for 18 min in stripping buffer (0.0626 M Tris–HCl, pH 6.7, 2% SDS, 0.1 M mercaptoethanol) and re-probed with a monoclonal mouse anti-beta-actin antibody (Sigma, St. Louis, MO, USA) as the loading control. The optical density of each specific band was measured using a computer-assisted imaging analysis system (GeneTools Match software; Syngene).

### 2.5. NHEJ Capacity of Peripheral Blood Lymphocytes from NPC Patients

To investigate the potential involvement of NHEJ in NPC development, we assessed the NHEJ capacity of peripheral blood lymphocytes established from the 43 NPC patients and correlated with risk genotypes [20,21]. Briefly, a plasmid pGL3 (Promega, Madison, WI, USA) was linearized using an *EcoRI* restriction enzyme and used for transfection into lymphocytes with various NHEJ genotypes using Lipofectamine 2000 (Invitrogen). After 48 h, the transfectants were harvested and assayed for luciferase activity, as previously described [20,21].

As for the neutral comet assay, peripheral blood lymphocytes were exposed to 100 μM of H_2_O_2_, post-incubated for 30 min or 24 h, trypsinized, washed, and re-suspended in ice-cold phosphate-buffered saline. Then, 10 μL of the cell suspension was embedded in the middle layer of 80–100−100 μL 3-layer low-melting-point agarose, and dried slides that were submersed for 1 h in the ice-cooled lysis buffer (2.5 M NaCl, 100 mM EDTA, 10 mM Tris–HCl, 1% Triton and 1% Na-laurylsarcosine, pH = 7.5). Slides were denatured and equilibrated for 30 min in the running buffer (90 mM Tris, 90 mM boric acid, 2 mM EDTA, pH = 7.5). Following the denaturation step, slides were electrophoresed at 0.8 V/cm for 25 min at 4 °C. Then, the slides were rinsed in ddH_2_O, fixed in 100% ethanol, and stained with 12.5 μL of 200X SYBR Green I.

The differences between the comet moment for the same patients with 30 min-treated or 24 h-treated H_2_O_2_ were calculated. Individual double-strand break repair capacity was defined by the formula: (Comet moment _30 min_ − Comet moment _24 h_)/Comet moment _30 min_ × 100%. The average for all the wild-type samples was set as 100% of the relative double-strand break repair capacity for the normalized comparisons of various genotypes.

### 2.6. Statistical Analysis Methodology

To verify that the controls were representative of the general population, the Hardy–Weinberg equilibrium was assessed using the goodness-of-fit test to determine the deviation of the genotype frequencies in the control group. The unpaired Student’s *t*-test was employed to compare the mean ages between the case and control groups. Pearson’s Chi-square test with Yates’ correction or Fisher’s exact test (when the number was less than 5) was used to compare the distribution of genotypes among subgroups. The comparisons of quantitative mRNA levels, protein levels, and NHEJ capacities between subgroups were performed using the unpaired Student’s *t*-test. A *p*-value of less than 0.05 was considered significant for all data. The odds ratios (ORs) and 95% confidence intervals (CIs) for NPC risk associated with genotypes were estimated using logistic regression.

## 3. Results

### 3.1. Demographic and Clinical Characteristics of Cases and Controls

Table 2 presents the frequency distributions of selected characteristics for the 208 NPC cases and 416 cancer-free controls. The controls were selected using frequency-matching, resulting in comparable distributions of gender and age between the cases and controls. Furthermore, the cases showed similar rates of smoking (40.9% vs. 38.0%, *p* = 0.5422), alcohol consumption (45.9% vs. 40.4%, *p* = 0.2399), and betel quid use (38.6% vs. 37.5%, *p* = 0.8840) when compared to the cancer-free controls (Table 2).

### 3.2. NPC Risk Associated with Individual NHEJ Genotypes

Table 3 summarizes the distributions of NHEJ genotypes and their associations with NPC risk, including *XRCC4* (rs6869366, rs3734091, rs28360071, rs28360317, rs1805377), *XRCC5* (rs828907, rs11685387, rs9288518), *XRCC6* (rs5751129, rs2267437, rs132770, rs132774), *XRCC7* rs7003908, and *Ligase4* rs1805388 genotypes, among NPC patients and controls. Significant associations with NPC risk were observed for three polymorphic sites.

First, for the *XRCC4* rs3734091 SNP, the controls had a frequency of 93.5% for the GG genotype, 6.3% for the GT genotype, and 0.2% for the TT genotype, whereas the NPC patients had a frequency of 87.5% for the GG, 11.1% for the GT, and 1.4% for the TT genotypes, respectively (Table 3). In logistic regression analyses, it was found that carriers of the heterozygous variant GT genotype had a significantly higher risk of NPC (OR = 1.89, 95%CI = 1.05–3.40, *p* = 0.0303), while the OR for carriers of the homozygous variant TT genotype was 4.27 (*p* for trend = 0.0465). In the dominant model, carriers of the GT + TT genotypes exhibited over a 2-fold increased risk of NPC (OR = 2.06, 95%CI = 1.17–3.63, *p* = 0.0170) compared to those with the wild-type GG genotype.

Second, for the *XRCC4* rs28360071 insertion/deletion (I/D) polymorphism, the frequency of II, ID, and DD genotypes was 67.3%, 28.6%, and 4.1% among the controls, and 54.8%, 37.0%, and 8.2% among the patients, respectively (Table 3). Carriers of the heterozygous variant ID and the homozygous variant DD genotypes had progressively increased risks of NPC with an OR of 1.59 (95% CI = 1.11–2.28) and 2.46 (95% CI = 1.21–4.98), respectively, compared to those with the wild-type II genotype (*p* for trend = 0.0045). In the dominant model, individuals carrying the ID + DD genotypes had a 1.7-fold (OR = 1.70, 95%CI = 1.21–2.39, *p* = 0.003) increased risk of NPC when compared to those with the II genotype.

Third, for the *XRCC6* rs5751129 SNP, the frequency of TT, CT, and CC genotypes was 80.5%, 17.6%, and 1.9% among the controls, and 67.8%, 26.4%, and 5.8% among the patients, respectively (*p* for trend = 0.0006, Table 3). Individuals carrying the heterozygous variant CT and homozygous variant CC genotypes exhibited progressively increased risks of NPC (OR = 1.79 and 3.56, 95% CI = 1.20–2.67 and 1.43–8.91, respectively) (*p* for trend = 0.0006). In the dominant model, carriers of the CT + CC genotypes had a nearly 2-fold increased risk of NPC (OR = 1.97, 95%CI = 1.35–2.87, *p* = 0.0006) compared to those with the TT genotype.

### 3.3. Combined Effects of NHEJ Genotypes on NPC Risk

We then examined the combined effects of the above three risk genotypes on the NPC risk (Table 4). The results showed that individuals carrying one risk genotype had a 2.49-fold increased risk (OR = 2.49, 95% CI = 1.69–3.67), those carrying two risk genotypes had a 1.98-fold increased risk (OR = 1.98, 95% CI = 1.24–3.16), while those carrying all three risk genotypes had a 6.28-fold higher risk of NPC (OR = 6.28, 95% CI = 1.84–21.43), although this risk estimate may be inflated due to the small numbers of subjects.

### 3.4. Genotype–Phenotype Correlation Analyses

**Figure 2:**
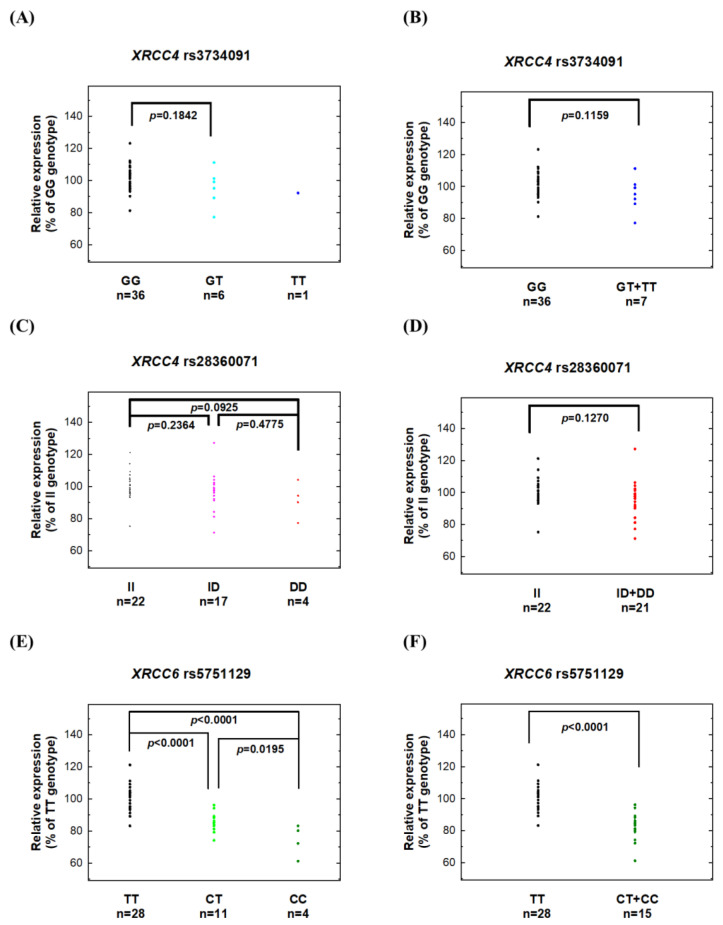
mRNA expression of XRCC4 and XRCC6 genes in adjacent normal tissues from NPC patients with different genotypes at three polymorphic sites: (**A**,**B**) *XRCC4* rs3734091, (**C**,**D**) *XRCC4* rs28360071, and (**E**,**F**) *XRCC6* rs5751129. The fold changes in expression were normalized using the GAPDH expression levels, and each assay was performed in triplicate.

**Figure 3:**
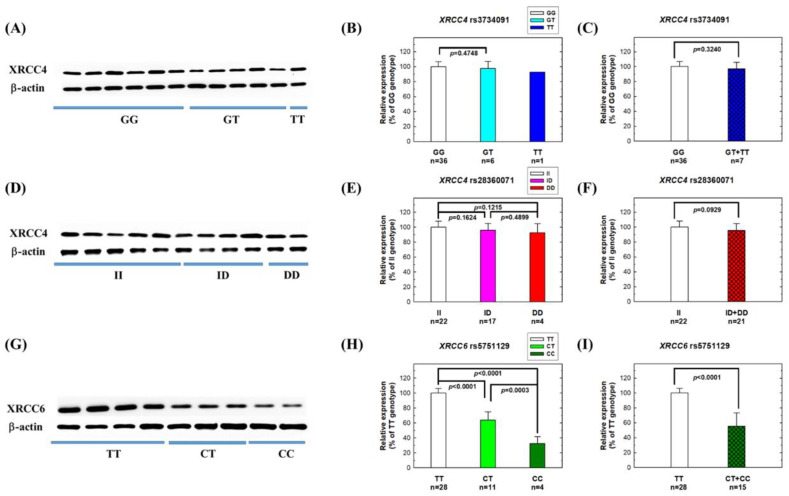
Protein expression levels of XRCC4 and XRCC6 in adjacent normal tissues from NPC patients with different genotypes at three polymorphic sites: (**A**–**C**) XRCC4 rs3734091, (**D**–**F**) XRCC4 rs28360071, and (**G**–**I**) XRCC6 rs5751129. Western blot images of proteins in tissues with different genotypes are presented in panels (**A**,**D**,**G**). Panels (**B**,**C**,**E**,**F**,**H**,**I**) show the fold changes of XRCC4 or XRCC6 protein, normalized to β-actin, in different risk genotypes as compared to the wild-type genotypes.

Next, we investigated the potential correlations between risk genotypes of *XRCC4* and *XRCC6* and their corresponding mRNA and protein expression levels. Among the 43 NPC patients, 36 had GG genotypes, 6 had GT genotypes, and only 1 had TT genotypes at *XRCC4* rs3734091. There appeared to be reduced XRCC4 mRNA (Figure 2B) and protein (Figure 3C) levels in patients with the risk genotypes (GT + TT), but the difference did not reach statistical difference (*p* = 0.1159 and 0.3240 for mRNA and protein, respectively), likely due to the small number of variant genotypes. Similarly, for *XRCC4* rs3734071, among the 43 NPC patients, 22 had II, 17 had ID, and 4 had DD genotypes, and there was a trend of reduced mRNA and protein expression levels in patients with the risk genotypes (ID + DD) compared to those with the wild-type II genotype (*p* = 0.1270 for mRNA comparison, Figure 2D, and *p* = 0.0929 for protein comparison, Figure 3F).

Most notably, for *XRCC6* rs5751129, the levels of mRNA and protein were significantly lower in patients carrying one risk allele (CT) (n = 11), and the lowest in patients carrying two risk alleles (CC) (n = 4), compared to those with the wild-type TT genotype (n = 28) (Figure 2E and Figure 3H). When we combined the risk genotypes (CT and CC) and compared them with the wild-type TT genotype, carriers of the risk genotypes had remarkably reduced mRNA and protein levels compared to those with the wild-type genotype (*p* < 0.0001 for both mRNA and protein, Figure 2F and Figure 3I).

### 3.5. Effects of Risk XRCC4 and XRCC6 Genotypes on NHEJ Repair Capacity

**Figure 4:**
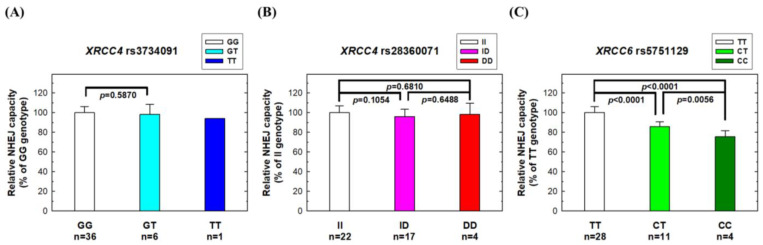
NHEJ repair capacity in peripheral blood lymphocytes from NPC patients with different genotypes at three polymorphic sites: (**A**) *XRCC4* rs3734091, (**B**) *XRCC4* rs28360071, and (**C**) *XRCC6* rs5751129. The host-cell reactivation assay was conducted in peripheral blood lymphocytes from NPC patients using a luciferase reporter plasmid.

**Figure 5:**
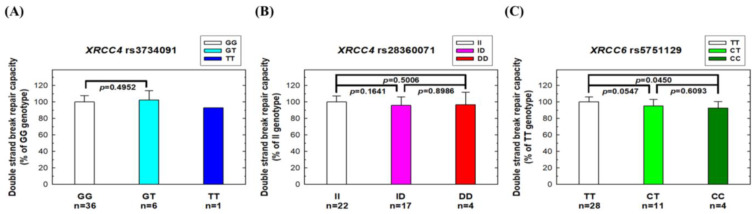
NHEJ repair capacity in peripheral blood lymphocytes by using the neutral comet assay from NPC patients with different genotypes at three polymorphic sites: (**A**) *XRCC4* rs3734091, (**B**) *XRCC4* rs28360071, and (**C**) *XRCC6* rs5751129.

Finally, we investigated the impact of risk *XRCC4* and *XRCC6* genotypes on the NHEJ repair capacity using peripheral blood lymphocytes from 43 NPC patients. No significant difference in NHEJ repair capacity was observed for those carrying various genotypes at the *XRCC4* rs3734091 or rs28360071 sites (Figure 4A,B). However, individuals with the risk genotypes (CT or CC) at *XRCC6* rs5751129 exhibited a significantly lower NHEJ repair capacity than those with the wild-type TT genotype (Figure 4C). No significant associations were observed between *XRCC4* genotypes and the DSB repair capacity, as measured by the neutral comet assay, but *XRCC6* rs5751129 genotypes were associated with the DSB repair capacity. The variant CT and TT genotypes had a lower DSB repair capacity than the wild-type CC genotype (*p* = 0.0547 and 0.0450, respectively) (Figure 5).

## 4. Discussion

Although NHEJ defects contribute to NPC pathogenesis, there have been few comprehensive evaluations of the NHEJ pathway in NPC patients using clinical samples. Genetic variations in specific NHEJ genes may be associated with altered risks of NPC. In 2015, we first reported the association of *XRCC6* rs5751129 with NPC risk in a small pilot study [12]. In that same study, we also measured XRCC6 mRNA and protein expression in 20 clinical samples. In this current study, we recruited a larger population (416 cases and 208 controls) and extended the investigation to 14 polymorphic sites in the 5 most important NHEJ genes. In addition to validating the significant role of *XRCC6* rs5751129 genotypes in NPC, we also provided more compelling evidence of the genotype–phenotype correlation for this SNP (Figure 2, Figure 3 and Figure 4). Additionally, we found that *XRCC4* rs3734091 or rs28360071 are novel NPC susceptibility loci (Table 3). There was no significant association for the other 11 investigated NHEJ polymorphic sites.

The roles of individual NHEJ genes in NPC etiology are not well-understood. In the current study, we found that at least two genes, *XRCC4* and *XRCC6*, are associated with NPC etiology. Although the impact of rs3734091 and rs28360071 on XRCC4 function in NPC patients is not yet fully understood, our mRNA and protein expression data suggest that these variants may have subtle effects on XRCC4 expression. XRCC4 forms a heterodimer with Ligase4 protein in the final NHEJ rejoining step [22]. XRCC4 enhances Ligase4 activity and acts as a bridge, linking Ligase4 to other NHEJ proteins, such as DNA-PK [23]. XRCC6 protein can form a heterodimer with XRCC5 protein or exist independently [24]. Our phenotypic data indicate that individuals carrying the variant genotypes (CT and CC) at *XRCC6* rs5751129, a SNP in the promoter region, had remarkably reduced expression levels of both mRNA and protein, resulting in a significantly lower NHEJ repair capacity, which could explain the increased NPC risk conferred by the variant genotypes. Furthermore, multiple risk alleles in NHEJ genes, such as *XRCC4* and *XRCC6*, can act synergistically to elevate a person’s risk of NPC (Table 4).

The lack of significant associations between NPC risk and other NHEJ genes, such as *XRCC5*, *XRCC7*, and *Ligase4*, does not mean that these genes are not involved in NPC etiology. These SNPs may not affect gene function and there might be other polymorphic sites on these genes that impact NPC risk. Further studies are needed to investigate other polymorphic sites and the biological interactions among the complex NHEJ machinery. On the other hand, although NHEJ is the major and “default” pathway for repairing DSB in mammalian cells, there is cross-talk, competition, and compensation between NHEJ and HR. When NHEJ is inhibited, HR can increase to compensate DSB activity [25]. Therefore, if one of the NHEJ genes has a genetic variant that causes subtle NHEJ function changes, it may not be detectable in our in vitro assays since they are not specifically measuring NHEJ activity. This may explain the lack of significant associations between XRCC4 genotypes and the DNA repair capacity.

No significant interaction was observed between age, gender, smoking, alcohol drinking, and betel quid chewing and NHEJ genotypes on NPC susceptibility (supplementary Tables S1–S3). This could be attributed to the limited sample size and lack of statistical power for the interaction analysis. Additionally, the DNA damages caused by these lifestyle factors are mainly not double-strand breaks and thus are not repaired primarily by the NHEJ pathway.

NHEJ deficiency may not only be involved in NPC etiology but also have important clinical implications. Targeting the DNA repair pathway can enhance the efficacy of DNA-damaging therapy (e.g., chemotherapy and radiotherapy) [26]. In particular, radiotherapy is the primary treatment for patients with NPC, and approximately 20% of patients’ experience treatment failure due to tumor radio-resistance. NHEJ-impaired patients, for example, those with the variant genotypes of XRCC6 rs5751129, may be more sensitive to radiotherapy, and agents and molecules that target NHEJ pathway proteins have been explored to enhance radiosensitivity and suppress radio-resistance [27,28]. Modulating the NHEJ pathway has significant clinical potential in NPC.

The present study has a few limitations. First, the sample size is limited for stratified and interaction analyses, especially for those polymorphisms with low variant genotype frequencies. Additionally, the use of adjacent normal tissues from only 43 NPC patients resulted in very few patients with a homozygous variant genotype, which hindered our ability to detect significant differences in mRNA and protein expressions, as well as NHEJ capacity, among patients with various genotypes. Second, since information on virus infection (e.g., Epstein–Barr virus and human papillomavirus) was not available, we were not able to adjust for this risk factor for NPC. Third, we were not able to assess the prognostic roles of these polymorphisms because the follow-up data on the survival status of NPC patients were insufficient. Finally, the generalizability of our findings to other populations needs to be validated by other populations.

In summary, our results suggested that genetic polymorphisms in *XRCC4* and *XRCC6* are associated with increased risks of NPC. Furthermore, individuals with lower mRNA and protein levels of XRCC4 and XRCC6 may have a lower NHEJ capacity and a higher risk of developing NPC.

## Figures and Tables

**Table 1:** Summary of the polymorphic sites, paired primer sequences, restriction enzymes, and DNA fragments after enzyme digestions for the polymorphic sites.

Genes	Polymorphic Sites	Primer Sequences (5′→3′)	Restriction Enzymes	Genetic Variants	DNA Fragments, bp	References
*XRCC4*	rs3734091	Forward: GCTAATGAGTTGCTGCATTTTA Reverse: TTTCTAGGGAAACTGCAATCTGT	*BbsI*	C A	308 204 + 104	[15]
	rs6869366	Forward: GATGCGAACTCAAAGATACTGA Reverse: TGTAAAGCCAGTACTCAAACTT	*HincII*	T G	300 200 + 100	[16]
	rs28360317	Forward (insertion): ATACTGTGTTTGGAACTCCT Forward (deletion): ATACTGTGTTTGGAACTAGA Reverse: TATCCTATCATCTCTGGATA		Insertion Deletion	239 No product	[16]
	rs1805377	Forward: TTCACTTATGTGTCTCTTCA Reverse: AACATAGTCTAGTGAACATC	*Tsp509I*	G A	237 158 + 79	[16]
	rs28360071	Forward: TCCTGTTACCATTTCAGTGTTAT Reverse: CACCTGTGTTCAATTCCAGCTT		Insertion Deletion	139 109	[16]
*XRCC5*	rs828907	Forward: TAGCTGACAACCTCACAGAT Reverse: ATTCAGAGGTGCTCATAGAG	*BfaI*	G T	252 171 + 81	[17]
	rs11685387	Forward: TCTAACTCCAGAGCTCTGAC Reverse: AACTCTGAGCATGCGCAGAT	*SpeI*	C T	311 203 + 108	[17]
	rs9288518	Forward: GGTGTGAAGACCTATCAATC Reverse: TTACAGAACAAGCCTTGCAC	*BsrI*	A G	275 165 + 110	[17]
*XRCC6*	rs5751129	Forward: TCATGGACCCACGGTTGTGA Reverse: CAACTTAAATACAGGAATGTCTTG	*DpnII*	T C	301 200 + 101	[12]
	rs2267437	Forward: AACTCATGGACCCACGGTTGTGA Reverse: CAACTTAAATACAGGAATGTCTTG	HaeII	C G	298 195 + 103	[12]
	rs132770	Forward: TACAGTCCTGACGTAGGAAG Reverse: AAGCGACCAACTTGGACAGA	*MnlI*	G A	226 146 + 80	[12]
	rs132774	Forward: GTATACTTACTGCATTCTGG Reverse: CATAAGTGCTCAGTACCTAT	*MscI*	TGG CCA	160 114 + 46	[12]
*XRCC7*	rs7003908	Forward: TGGTGCTCAGCTTCTGGCTT Reverse: CATCCCTGCCAGCTCTTCTG	*TaqI*	T G	301 235 + 66	[18]
*Ligase4*	rs1805388	Forward: TCTGTATTCGTTCTAAAGTTGAACA Reverse: TGCTTTACTAGTTAAACGAGAAGAT	*HpyCH4III*	A G	121 65 + 56	[19]

**Table 2:** Distributions of selected characteristics of the cases and controls.

Characteristic	Controls (n = 416)			Patients (n = 208)			*p*-Value
	n	%	Mean (SD)	n	%	Mean (SD)	
Age (years)			49.9 (11.5)			50.6 (11.0)	0.4639 ^a^
Gender							
Male	306	73.6%		153	73.6%		
Female	110	26.4%		55	26.4%		1.0000 ^b^
Smoking status							
Ever smokers	158	38.0%		85	40.9%		
Non-smokers	258	62.0%		123	59.1%		0.5422 ^b^
Drinking status							
Ever drinkers	168	40.4%		95	45.9%		
Non-drinkers	248	59.6%		113	54.1%		0.2399 ^b^
Betel quid status							
Ever chewers	156	37.5%		80	38.6%		
Non-chewers	260	62.5%		128	61.4%		0.8840 ^b^

^a^ Based on the unpaired Student’s *t*-test. ^b^ Based on the Chi-square test with Yates’ correction.

**Table 3:** Distributions of NHEJ genotypes among the NPC patients and controls and the associations of genotypes with NPC risk.

Genotype	Controls		Patients		OR (95% CI)	*p*-Value
	n	%	n	%		
***XRCC4***						
rs6869366						
TT	391	94.0%	192	92.3%	1.00 (reference)	
GT	25	6.0%	16	7.7%	1.30 (0.68–2.50)	0.5298
						
rs3734091						
GG	389	93.5%	182	87.5%	1.00 (reference)	
GT	26	6.3%	24	11.1%	**1.89 (1.05–3.40)**	0.0303 *
TT	1	0.2%	2	1.4%	4.27 (0.39–47.45)	0.5043
*p*-value for trend						0.0465 *
GT + TT					**2.06 (1.17–3.63)**	0.0170 *
						
rs28360071						
II	280	67.3%	114	54.8%	1.00 (reference)	
ID	119	28.6%	77	37.0%	**1.59 (1.11–2.28)**	0.0148 *
DD	17	4.1%	17	8.2%	**2.46 (1.21–4.98)**	0.0181 *
*p*-value for trend						0.0045 *
ID + DD					**1.70 (1.21–2.39)**	0.0030 *
						
rs28360317						
II	248	59.6%	120	57.7%	1.00 (reference)	
ID	140	33.7%	70	33.7%	1.03 (0.72–1.48)	0.9312
DD	28	6.7%	18	8.6%	1.33 (0.71–2.50)	0.4723
*p*-value for trend						0.6762
ID + DD					1.08 (0.77–1.52)	0.7084
						
rs1805377						
AA	216	51.9%	111	53.3%	1.00 (reference)	
AG	172	41.4%	85	40.9%	0.96 (0.68–1.36)	0.8942
GG	28	6.7%	12	5.8%	0.83 (0.41–1.70)	0.7478
*p*-value for trend						0.8769
AG + GG					0.94 (0.68–1.32)	0.7987
						
***XRCC5***						
rs828907						
GG	268	64.4%	128	61.5%	1.00 (reference)	
GT	125	30.1%	66	31.7%	1.11 (0.77–1.59)	0.6564
TT	23	5.5%	14	6.8%	1.27 (0.63–2.56)	0.6169
*p*-value for trend						0.7233
GT + TT					1.16 (0.82–1.63)	0.4457
						
rs11685387						
TT	234	56.3%	120	57.7%	1.00 (reference)	
CT	147	35.3%	70	33.7%	0.93 (0.65–1.33)	0.7548
CC	35	8.4%	18	8.6%	1.00 (0.55–1.85)	0.9927
*p*-value for trend						0.9171
CT + CC					0.94 (0.67–1.32)	0.7971
						
rs9288518						
GG	229	55.0%	120	57.7%	1.00 (reference)	
AG	150	36.1%	73	35.1%	0.93 (0.65–1.33)	0.4985
AA	37	8.9%	15	7.2%	0.77 (0.41–1.47)	0.5280
*p*-value for trend						0.7116
AG + AA					0.90 (0.64–1.26)	0.5881
						
***XRCC6***						
rs5751129						
TT	335	80.5%	141	67.8%	1.00 (reference)	
CT	73	17.6%	55	26.4%	**1.79 (1.20–2.67)**	0.0058 *
CC	8	1.9%	12	5.8%	**3.56 (1.43–8.91)**	0.0084 *
*p*-value for trend						0.0006 *
CT + CC					**1.97 (1.35–2.87)**	0.0006 *
						
rs2267437						
CC	276	66.3%	134	64.4%	1.00 (reference)	
CG	123	29.6%	67	32.2%	1.12 (0.78–1.61)	0.5962
GG	17	4.1%	7	3.4%	0.85 (0.34–2.09)	0.8940
*p*-value for trend						0.7468
CG + GG					1.09 (0.77–1.54)	0.6983
						
rs132770						
GG	315	75.7%	158	76.0%	1.00 (reference)	
AG	89	21.4%	41	19.7%	0.92 (0.61–1.39)	0.7678
AA	12	2.9%	9	4.3%	1.50 (0.62–3.62)	0.5090
*p*-value for trend						0.5925
AG + AA					0.99 (0.67–1.46)	0.9473
						
rs132774						
GG	329	79.1%	171	82.2%	1.00 (reference)	
CG	79	20.9%	37	17.8%	0.82 (0.53–1.25)	0.7161
						
***XRCC7***						
rs7003908						
TT	209	50.2%	112	53.8%	1.00 (reference)	
GT	175	42.1%	83	39.9%	0.89 (0.63–1.25)	0.5485
GG	32	7.7%	13	6.3%	0.76 (0.38–1.50)	0.5305
*p*-value for trend						0.6353
GT + GG					0.87 (0.62–1.21)	0.4445
						
***Ligase4***						
rs1805388						
CC	235	56.5%	112	53.8%	1.00 (reference)	
CT	148	35.6%	79	38.0%	1.12 (0.79–1.60)	0.5911
TT	33	7.9%	17	8.2%	1.08 (0.58–2.02)	0.9348
*p*-value for trend						0.8168
CT + TT					1.11 (0.80–1.56)	0.5883

OR: odds ratio, CI: confidence interval. *p*-values for genotypes were calculated by the Chi-square test with Yates’ correction. *P*_trend_: *p*-value for trend analysis, *: *p* < 0.05.

**Table 4:** Combined effects of NHEJ genotypes on NPC risk.

# of Risk Genotypes	Controls, n	Cases, n	OR (95%CI)	*p*-Values
0	245	78	1.00 (Reference)	
1	102	81	**2.49 (1.69–3.67)**	0.0001 *
2	65	41	**1.98 (1.24–3.16)**	0.0055 *
3	4	8	**6.28 (1.84–21.43)**	0.0029 *
*p*_trend_				0.0001 *

OR: odds ratio, 95%CI: 95% confidence interval, *p*_trend_: *p*-value by trend analysis. The GT or TT genotypes of *XRCC4* rs3734091, the ID or DD genotypes of *XRCC4* rs28360071, and the CT or CC genotypes of *XRCC6* rs5751129, were denoted as risk genotypes. *p*-values were calculated by the 2 × 4 chi-square test, *: *p* < 0.05.

## Data Availability

The genotyping results and clinical data supporting the findings of this study are available from the corresponding authors upon reasonable requests via email at artbau2@gmail.com.

## Supplementary Materials

The following supporting information can be downloaded at: https://www.mdpi.com/article/10.3390/biomedicines11061648/s1, Table S1: Associations between *XRCC4* rs3734091 genotypes and NPC risk in stratified analyses; Table S2: Associations between *XRCC4* rs28360071 genotypes and NPC risk in stratified analyses; Table S3: Associations between *XRCC6* rs5751129 genotypes and NPC risk in stratified analyses.
