# Supplementary material for: Non-Homologous End-Joining Pathway Genotypes Significantly Associated with Nasopharyngeal Carcinoma Susceptibility
Source: Biomedicines. 2023 Jun 6;11(6):1648. doi: 10.3390/biomedicines11061648 (PMC10296066; doi:10.3390/biomedicines11061648)
Supplement: Supplementary file 1 [file biomedicines-11-01648-s001.zip › biomedicines-2399414-supplementary.pdf]

Supplementary Table S1. Associations between *XRCC4* rs3734091 genotypes and NPC risk in stratified analyses

Genotype	Controls	Cases	aOR (95% CI)[a]	*p*-Value[b]
Age				
≤44 years old				
GG	196	87	1.00 (ref)	
GT	11	12	2.18 (0.98-4.86)	0.0600
TT	1	2	3.87 (0.53-41.75)	0.2294
>44 years old				
GG	193	95	1.00 (ref)	
GT	15	12	1.49 (0.74-2.83)	0.3224
Gender				
Males				
GG	288	135	1.00 (ref)	
GT	17	16	1.84 (0.83-3.72)	0.0791
TT	1	2	3.63 (0.37-42.68)	0.2432
Females				
GG	101	47	1.00 (ref)	
GT	9	8	1.78 (0.71-4.43)	0.3193
Smoking behaviors				
Smokers				
GG	147	75	1.00 (ref)	
GT	10	9	1.63 (0.64-3.95)	0.3463
TT	1	1	1.82 (0.31-27.36)	1.0000
Non-smokers				
GG	242	107	1.00 (ref)	
GT	16	15	1.94 (0.89-3.84)	0.0679
TT	0	1	---	0.3086
Alcohol drinking behaviors				
Drinkers				
GG	155	82	1.00 (ref)	
GT	12	12	1.77 (0.84-3.56)	0.2025
TT	1	1	1.72 (0.78-40.21)	1.0000
Non-drinkers				
GG	234	100	1.00 (ref)	
GT	14	12	1.79 (0.78-3.84)	0.1336
TT	0	1	---	0.3015
Betel quid chewing				
Chewers				
GG	147	71	1.00 (ref)	
GT	8	8	2.11 (0.71-4.83)	0.2504
TT	1	1	1.88 (0.14-28.98)	0.5484
Non-chewers				
GG	240	111	1.00 (ref)	
GT	18	16	1.88 (0.90-3.39)	0.1017
TT	0	1	---	0.3182

[a], by multivariate logistic regression analysis after the adjustments of confounding factors; [b], by chi-square with Yates' correction (n>5) or Fisher's exact test (n≤5); CI, confidence interval; aOR, adjusted odds ratio.

Supplementary Table S2. Associations between *XRCC4* rs28360071 genotypes and NPC risk in stratified analyses

Genotype	Controls	Cases	aOR (95% CI)[a]	*p*-Value[b]
Age				
≤44 years old				
II	125	60	1.00 (ref)	
ID	69	32	0.90 (0.65-1.44)	0.8968
DD	14	9	1.27 (0.73-2.95)	0.6828
>44 years old				
II	123	60	1.00 (ref)	
ID	71	38	1.05 (0.77-1.54)	0.8141
DD	14	9	1.40 (0.68-3.04)	0.7090
Gender				
Males				
II	185	90	1.00 (ref)	
ID	100	49	1.04 (0.78-1.69)	0.9735
DD	21	14	1.35 (0.74-2.21)	0.5040
Females				
II	63	30	1.00 (ref)	
ID	40	21	1.11 (0.70-1.95)	0.9167
DD	7	4	1.09 (0.42-3.87)	0.7837
Smoking behaviors				
Smokers				
II	95	48	1.00 (ref)	
ID	52	30	1.12 (0.64-1.91)	0.7547
DD	11	7	1.33 (0.55-3.77)	0.8532
Non-smokers				
II	153	72	1.00 (ref)	
ID	88	40	1.00 (0.58-1.82)	0.9788
DD	17	11	1.41 (0.67-2.96)	0.5748
Alcohol drinking behaviors				
Drinkers				
II	103	56	1.00 (ref)	
ID	53	31	1.12 (0.73-2.01)	0.9046
DD	12	8	1.21 (0.58-2.87)	0.8628
Non-drinkers				
II	145	64	1.00 (ref)	
ID	87	39	1.09 (0.57-1.68)	0.9494
DD	16	10	1.24 (0.70-2.95)	0.5567
Betel quid chewing				
Chewers				
II	96	45	1.00 (ref)	
ID	50	28	1.08 (0.72-1.99)	0.6534
DD	10	7	1.33 (0.44-4.57)	0.6209
Non-chewers				
II	152	75	1.00 (ref)	
ID	90	42	1.02 (0.71-1.79)	0.9034
DD	18	11	1.14 (0.49-2.86)	0.7517

[a], by multivariate logistic regression analysis after the adjustments of confounding factors; [b], by chi-square with Yates' correction (n>5) or Fisher's exact test (n≤5); CI, confidence interval; aOR, adjusted odds ratio.

Supplementary Table S3. Associations between *XRCC6* rs5751129 genotypes and NPC risk in stratified analyses

**Genotype**	**Controls**	**Cases**	**aOR (95% CI)[a]**	***p*-Value[b]**
Age				
≤44 years old				
TT	168	69	1.00 (ref)	
CT	36	26	1.55 (0.86-2.77)	0.0755
CC	4	6	3.37 (0.93-11.84)	0.0712
>44 years old				
TT	167	71	1.00 (ref)	
CT	37	29	1.79 (0.99-3.14)	0.0444*
CC	4	6	3.36 (0.93-10.78)	0.0741
Gender				
Males				
TT	243	107	1.00 (ref)	
CT	57	38	1.44 (0.81-2.53)	0.1062
CC	6	8	2.91 (0.98-7.06)	0.0712
Females				
TT	92	34	1.00 (ref)	
CT	16	17	1.61 (0.87-3.95)	0.0132*
CC	2	4	3.30 (0.67-19.48)	0.0569
Smoking behaviors				
Smokers				
TT	133	58	1.00 (ref)	
CT	23	22	1.79 (0.99-3.78)	0.0288*
CC	2	5	3.51 (0.97-19.64)	0.0345*
Non-smokers				
TT	202	83	1.00 (ref)	
CT	50	33	1.53 (0.89-2.54)	0.0889
CC	6	7	2.73 (0.87-7.46)	0.1119
Alcohol drinking behaviors				
Drinkers				
TT	133	65	1.00 (ref)	
CT	32	24	1.52 (0.82-2.98)	0.2186
CC	3	6	3.34 (0.68-12.88)	0.0657
Non-drinkers				
TT	202	76	1.00 (ref)	
CT	41	31	1.79 (0.98-3.32)	0.0148*
CC	5	6	3.04 (0.96-8.38)	0.1048
Betel quid chewing				
Chewers				
TT	129	55	1.00 (ref)	
CT	24	20	1.82 (0.91-3.14)	0.0726
CC	3	5	3.68 (0.92-13.69)	0.1107
Non-chewers				
TT	206	86	1.00 (ref)	
CT	49	35	1.68 (0.90-2.31)	0.0478*
CC	5	7	3.11 (0.94-8.57)	0.0706

[a], by multivariate logistic regression analysis after the adjustments of confounding factors; [b], by chi-square with Yates' correction (n>5) or Fisher's exact test (n≤5); [*], statistically significant; CI, confidence interval; aOR, adjusted odds ratio.
